# Biochemical and immunocytochemical characterization of coronins in platelets

**DOI:** 10.1080/09537104.2019.1696457

**Published:** 2019-12-04

**Authors:** David R. J. Riley, Jawad S. Khalil, Khalid M. Naseem, Francisco Rivero

**Affiliations:** 1 Centre for Atherothrombosis and Metabolic Disease, Hull York Medical School, Faculty of Health Sciences, University of Hull, Hull, UK; 2 School of Physiology, Pharmacology and Neuroscience, Faculty of Life Sciences, University of Bristol, Bristol, UK; 3 Leeds Institute for Cardiovascular and Metabolic Medicine, University of Leeds, Leeds, UK

**Keywords:** Actin cytoskeleton, actin nodule, Arp2/3 complex, collagen, coronin, platelets, thrombin, Triton insoluble pellet

## Abstract

Rapid reorganization of the actin cytoskeleton in response to receptor-mediated signaling cascades allows platelets to transition from a discoid shape to a flat spread shape upon adhesion to damaged vessel walls. Coronins are conserved regulators of the actin cytoskeleton turnover but they also participate in signaling events. To gain a better picture of their functions in platelets we have undertaken a biochemical and immunocytochemical investigation with a focus on Coro1. We found that class I coronins Coro1, 2 and 3 are abundant in human and mouse platelets whereas little Coro7 can be detected. Coro1 is mainly cytosolic, but a significant amount associates with membranes in an actin-independent manner and does not translocate from or to the membrane fraction upon exposure to thrombin, collagen or prostacyclin. Coro1 rapidly translocates to the Triton insoluble cytoskeleton upon platelet stimulation with thrombin or collagen. Coro1, 2 and 3 show a diffuse cytoplasmic localization with discontinuous accumulation at the cell cortex and actin nodules of human platelets, where all three coronins colocalize. Our data are consistent with a role of coronins as integrators of extracellular signals with actin remodeling and suggests a high extent of functional overlap among class I coronins in platelets.

## Introduction

Platelets are anucleate fragments of megakaryocytes that play pivotal roles in hemostasis, thrombosis, wound healing and immunological processes. Platelets display a remarkable morphological plasticity. While in circulation they have a characteristic discoid shape, but are capable of undergoing profound changes upon adhesion to damaged blood vessel walls, transitioning to a spherical shape that extends filopodia and lamellipodia as the cell spreads and flattens [1]. This process is accompanied by secretion of granules and activation of integrins that support and consolidate the formation of a platelet aggregate. Remodeling of the cytoskeleton, formed by a network of actin filaments and a marginal ring of microtubules and associated proteins constitutes a crucial aspect of platelet function and is the result of multiple exquisitely integrated signaling cascades [2]. A plethora of proteins with various biochemical activities is responsible for the dynamics of actin remodeling during platelet activation, including actin nucleators like formins and the Arp2/3 complex and their regulators (WAVE, WASP), monomeric actin-binding proteins like profilin, β-thymosin and the cyclase-associated protein (CAP) and others like gelsolin, cofilin, and coronins [3–5].

Coronins constitute a family of conserved regulators of the actin cytoskeleton turnover. The defining architectural element of this family is the WD40 repeat that folds in a β-propeller structure and characteristically participates in protein–protein interactions [6]. The β-propeller is flanked by short highly conserved extensions. The C-terminal extension is followed by a variable unique region and a coiled-coil domain, and the latter involved in oligomerization [7,8]. Mammals express seven coronins that have been grouped into three classes [9,10]. Among class I coronins (Coro1, 2, 3 and 6), Coro1 is the most widely studied for its role in coordinating actin dynamics through modulation of Arp2/3 complex and cofilin function [11]. Coro1 also plays less well-understood roles in NADPH oxidase complex regulation, calcium release, vesicle trafficking and apoptosis [12–15]. Class I coronins localize at the leading edge of migrating cells and to phagosomes in neutrophils [7,15,16]. Class II coronins (Coro4 and 5) are involved in focal adhesion turnover, reorganization of the cytoskeleton and cell migration [17,18]. The class III coronin (Coro7) has an unusual structure, as it consists of two coronin blocks in tandem and lacks a coiled-coil region. This atypical coronin plays a role in Golgi morphology maintenance and does not appear to participate in actin-related processes [19].

While coronins have been widely investigated in a variety of cell types, very little is known about these proteins in platelets. A recent report investigating the role of Coro1 in platelet function using a knockout mouse model revealed impaired agonist-induced actin polymerization and cofilin phosphoregulation and altered thrombus formation in vivo as salient phenotypes, in the absence of an overt hemostasis defect in vivo [5]. This mild phenotype suggests a complex picture, with class I coronins potentially sharing roles extensively in platelets.

We have undertaken a biochemical and immunocytochemical investigation as an approach toward a clearer picture of the functions of coronins in platelets. We show that class I coronins are abundant in human and mouse platelets whereas little Coro7 can be detected. Coro1 is mainly cytosolic, but a significant amount associates with membranes in an actin-independent manner and does not translocate from or to the membrane fraction upon platelet stimulation. In immunocytochemistry studies, Coro1, 2 and 3 show a diffuse cytoplasmic localization with accumulation at the cell cortex and actin nodules, where all three coronins colocalize. Our study strengthens the view of complex redundancy among coronins in platelets, an aspect to take into consideration in future functional studies.

## Materials and Methods

### Reagents

Primary antibodies against following proteins were used: Coro1 (ab56820 and ab72212), Coro2 (ab99407), CAP1 (ab133655), β-actin (ab20272) from Abcam (Cambridge, UK); Coro3 (K6-444 hybridoma supernatant) [7], Coro7 (K37-142-1 hybridoma supernatant)[20]; CD36 (H-300 sc-9154), Syk (4D10 sc-1240), β3-integrin (HC93 sc-14009) and Gαs (sc-823) from Santa Cruz Biotechnology (Heidelberg, Germany); cofilin (D3F9 #5175), profilin-1 (#3237), phosphor-VASP (Ser157) (#3111) and phosphor-MLC (Ser19) (#3671) from Cell Signaling Technology (Leiden, The Netherlands); α-tubulin (05–829) and GAPDH (6C5-CB1001) from Calbiochem/Merck (Watford, UK); p34-Arc/ARPC2 (07–227) from Millipore/Merck; vinculin (SAB4200080) from Sigma/Merck; Myc, mouse monoclonal 9E10 (kind gift of Angelika A. Noegel, University of Cologne, Germany). Specificity of antibodies raised against Coro1, Coro2 and Coro3 was tested on recombinantly expressed proteins in HEK 293T cell lysates (Supplemental Figure 1)

**Figure 1.**
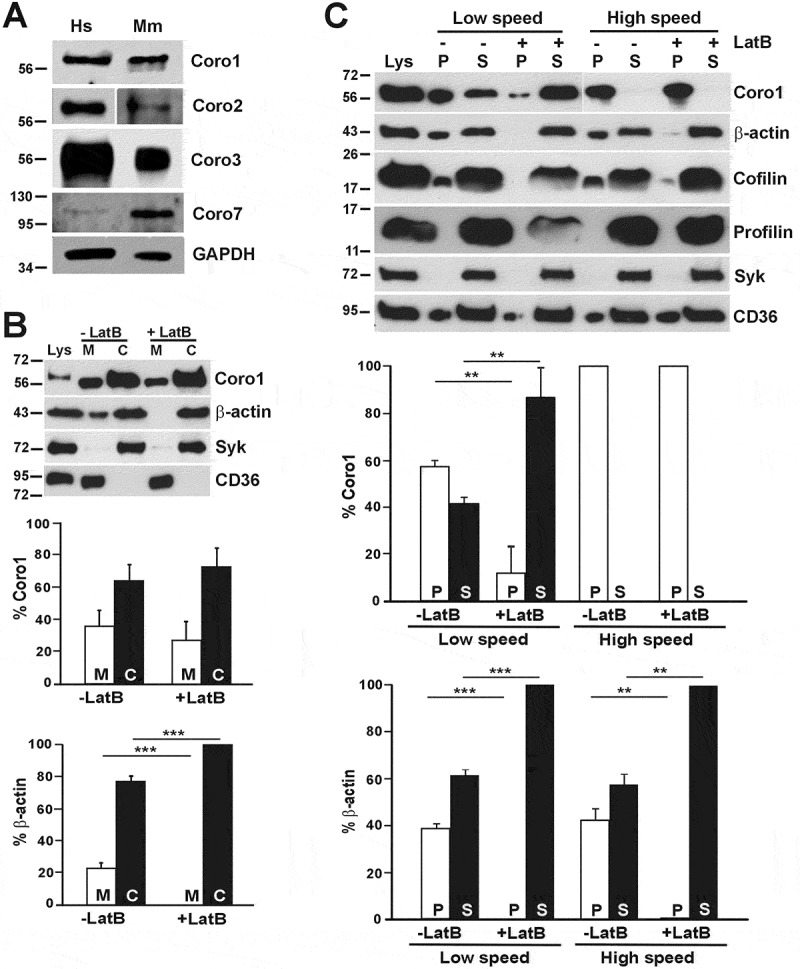
Coronins present in human and mouse platelets and subcellular distribution of human Coro1. (A) Western blot of human and mouse platelet lysates. Twenty micrograms of protein were resolved by 10% SDS-PAGE, blotted onto PVDF membrane and probed with antibodies for the indicated proteins. The mouse Coro2 blot corresponds to a higher exposure than the human one. The Coro7 blot was enhanced to make the human protein apparent (see Supplemental Figure 2 for details). GAPDH was used as a loading control. (B) Subcellular fractionation. Human platelets were lysed by freeze-thaw in liquid nitrogen and spun at 100,000 × g for 1 h to separate membrane (M) and cytosolic (C) fractions. The fractions were normalized by volume and resolved by 12% SDS-PAGE, blotted onto PVDF membrane and probed with antibodies for the indicated proteins. CD36 was used as a membrane marker and Syk as a cytosolic marker in resting platelets. Latrunculin B (LatB; 20 µM, 20 min) was used to depolymerize F-actin prior to lysis. Coro1 and actin distribution were quantified by densitometry and expressed as a percentage relative to the respective totals (M + C). (C) Association of Coro1 to actin in the detergent-insoluble pellet. Human platelets (8 × 10^8^/ml) were lysed in the presence of 1% Triton X-100 and lysates spun at low speed (15,600 × g) for 20 min and high speed (100,000 × g) for 1 h. Supernatant (S) and pellet (P) fractions were normalized by volume and resolved by 12% SDS-PAGE, blotted onto PVDF membrane and probed with antibodies for the indicated proteins. LatB (20 µM, 20 min) was used to depolymerize F-actin prior to lysis. Coro1 and actin distribution in pellet and supernatant were quantified by densitometry and expressed as a percentage of the respective total (P + S). Data of B and C represent mean ± SD of three independent experiments.‬‬‬‬‬‬‬‬‬‬‬‬‬‬‬‬‬‬‬‬‬‬‬‬‬‬‬‬‬‬‬‬‬‬‬‬‬‬‬‬‬‬‬‬‬‬‬‬‬‬‬‬‬‬‬‬‬‬‬‬‬‬‬‬‬‬‬‬‬‬‬ **P< .01, ***P < .001 vs LatB-treated, Student’s t-test.

Secondary antibodies Alexa Fluor 568- or 488-conjugated anti-mouse and anti-rabbit immunoglobulins (Molecular Probes, Thermo Fisher Scientific, Altrincham, UK) were used for immunofluorescence. Peroxidase-conjugated anti-mouse and anti-rabbit immunoglobulins (Merck) or IRDye 680 or IRDye 800 anti-mouse and anti-rabbit immunoglobulins (LI-COR Biosciences, Lincoln, USA) were used for Western blot.

Human fibrinogen was from Enzyme Research (Swansea, UK), collagen (Kollagenreagens Horm) was from Takeda (Osaka, Japan), latrunculin B was from Enzo Life Sciences (Exeter, UK), nocodazole and CK-666 were from Tocris Bioscience (Abingdon, UK). PGI2 was from Cayman Chemical (Michigan, USA). Thrombin, FITC or TRITC-conjugated phalloidin were from Merck. Alexa Fluor 680-conjugated phalloidin was from Thermo Fisher Scientific. Other reagents were from Merck unless otherwise indicated.

### Human Platelet Preparation

Human blood was taken from drug-free volunteers by clean venepuncture into acid citrate dextrose (ACD) (29.9 mM trisodium citrate, 113.8 mM glucose, 72.6 mM NaCl and 2.9 mM citric acid, pH 6.4). Platelet-rich plasma (PRP) was obtained by centrifugation of whole blood at 190 × g for 15 min at room temperature. Platelets were isolated from PRP by centrifugation at 800 × g for 12 min in the presence of 6 mM citric acid. Platelets were washed in pH 6.5 buffer (0.036 M citric acid, 0.01 M EDTA, 0.005 M glucose, 0.005 M KCl, 0.09 M NaCl) and centrifuged at 800 × g for 12 min. Sedimented platelets were resuspended in modified Tyrode’s buffer (150 mM NaCl, 5 mM HEPES, 0.55 mM NaH_2_PO_4_, 7 mM NaHCO_3_, 2.7 mM KCl, 0.5 mM MgCl_2_, and 5.6 mM glucose, pH 7.4) and maintained at 37°C for 30 min prior to experiments. The study was approved by the Hull York Medical School Research Ethics Committee and all research was performed in accordance with relevant guidelines and regulations. Informed consent was obtained from all blood donors.

### Mouse Platelet Preparation

Blood was taken by cardiac puncture into ACD, centrifuged at 100 × g for 5 min and the PRP was collected in a separate tube. Modified Tyrode’s buffer was added to the blood and the procedure repeated to increase the platelet yield. The platelets were then pelleted at 800 × g for 6 min, resuspended in modified Tyrode’s buffer and maintained at 37°C for 30 min prior to experiments.

### Platelet Fractionation

Washed platelet suspensions (5 × 10^8^ platelets/ml), either untreated or treated with various substances for the appropriate time, were mixed with an equal volume of fractionation buffer (320 mM sucrose, 4 mM HEPES, 0.5 mM Na_3_VO_4_, pH 7.4) supplemented with phosphatase and protease inhibitor cocktail. Latrunculin B (LatB) was used at 20 µM for 20 min to depolymerize F-actin prior to lysis. Samples were subjected to five freeze-thaw cycles in liquid nitrogen. Intact platelets were removed by centrifugation at 1,000 × g for 5 min at 4°C and fractionation was done by centrifugation at 100,000 × g for 60 min at 4°C. The fractions were normalized by volume and analyzed by Western blot.

### Detergent-Insoluble Pellet Extraction

Washed platelet suspensions (1 × 10^9^ platelets/ml) were lysed in an equal volume of Triton X-100 containing lysis buffer (2% Triton X-100, 10 mM Tris-HCl, 10 mM EGTA, pH 7.4) supplemented with protease inhibitors. Lysates were spun at 15,600 × g for 20 min (low speed) or 100,000 × g for 1 h (high speed) to separate the detergent soluble fraction from the detergent-insoluble pellet. The fractions were normalized by volume, resolved on 10% SDS-PAGE and analyzed by Western blot.

### Immunoprecipitation

Platelets (1x10^9^/ml) were lysed with one volume of lysis buffer (20 mM HEPES, 30 mM NaCl, 0.3 mM EDTA, 2% n-dodecyl β-D-maltoside, 0.5 mM DTT, pH 7.4) supplemented with protease inhibitors for 30 min on ice. Two hundred to five hundred micrograms per milliliter of protein lysate were incubated overnight with gentle rotation at 4°C with 1 μg of specific antibody or same species control immunoglobulin. Twenty microliters of pre-equilibrated protein G Sepharose beads were added to lysate-antibody mixture and incubated at 4°C for 1 h. After several washing steps with TBS-T (20 mM Tris-HCl, 150 mM NaCl, 0.1% Tween 20, pH 7.4) the beads were resuspended in 2× Laemmli buffer and immunocomplexes analyzed by Western blot.

### Western Blot

Proteins were resolved by SDS-polyacrylamide gel electrophoresis (PAGE) and blotted onto polyvinylidene difluoride (PVDF) membrane. The membrane was incubated with the relevant primary antibody and either the corresponding peroxidase-conjugated secondary antibody followed by enhanced chemiluminescence detection (Pierce, Thermo Fisher Scientific Inc.) or the corresponding fluorochrome-labeled secondary antibody and visualized and quantified with an LI-COR Odyssey CLx Imaging System (LI-COR Biosciences, Lincoln, USA).

### Immunostaining and Microscopy

Washed platelets in suspension were fixed with an equal volume of ice-cold 4% paraformaldehyde (PFA) in PBS or, for tubulin staining, 3% PFA in 16 mM PIPES, 0.2 mM MgCl_2_, 0.2 mM EGTA, pH 6.8 and spun at 350 × g for 10 min on poly-L-lysine (0.01% in PBS) coated coverslips. For adhesion studies, coverslips were coated overnight at 4°C with 100 µg/ml fibrinogen or collagen and blocked with heat-denatured fatty-acid-free bovine serum albumin for 1 h before the experiment. Washed platelets were allowed to spread for 45 min at 37°C, then fixed with 4% PFA. Fixed platelets were permeabilized with 0.3% Triton® X-100 in PBS for 5 min and stained for 1 h at room temperature with the indicated primary antibodies followed the corresponding secondary antibodies and/or fluorescently labeled phalloidin diluted in PBG (0.5% BSA, 0.05% fish gelatin in PBS). Platelets were imaged by fluorescence microscopy using a Zeiss ApoTome.2 equipped with AxioCam 506 and Zeiss Plan-Apochromat 63×/1.4 and 100×/1.4 oil immersion objectives. Images were processed with Zeiss Zen software.

### Statistical Analysis

Experimental data were analyzed by GraphPad Prism v6.0 (La Jolla, CA, USA). Data are presented as means ± standard error of the mean (SEM) or standard deviation (SD) of at least three independent experiments. Normality was assessed by the Shapiro-Wilk test. Differences between groups were assessed using the Student’s t-test, Mann-Whitney U-test, Wilcoxon test, analysis of variance (ANOVA) or Kruskal-Wallis test and statistical significance taken at p ≤ 0.05.

## Results

### Platelets Express at Least Four Coronins

Proteomics and transcriptomics studies indicate that both human and mouse platelets express Coro1, 2, 3 and 7, while other coronins are practically undetectable (Supplemental Table 1 and 2). To demonstrate the presence of coronins in platelets we resolved human and mouse platelet lysates by SDS-PAGE, followed by western blot with a panel of antibodies specific for various coronins (see Supplemental Figure 1 for antibody specificity). Coro1, 2 and 3 appeared as single bands with apparent molecular weights of or above 56 kDa whereas Coro7 appeared as a single band of 100 kDa (Figure 1A). While Coro1, 2 and 3 appear relatively abundant, Coro 7 is expressed at much lower levels in both human and mouse platelets. In this study we will mainly focus on human Coro1 as a paradigm of class I coronins, but will also address Coro3 and Coro2 is some assays and will verify if our findings apply to mouse coronins.

### Subcellular Distribution of Coro1

To investigate the distribution of Coro1 we carried out a simple subcellular fractionation in human platelets. Resting platelets were lysed in an isotonic sucrose solution and cytosol and membrane fractions separated by ultracentrifugation and analyzed by immunoblot. As shown in Figure 1B, most of Coro1 (64%) was recovered in the cytosolic fraction and the rest associated with the membrane fraction. The blot was reprobed for β-actin and 77% of the actin was cytosolic and the rest membrane-associated. Since Coro1 is an actin-binding protein, we further investigated whether this membrane association is mediated by actin. Resting platelets were treated with 20 μM latrunculin B (LatB) to depolymerize F-actin prior to subcellular fractionation. As expected, under these conditions almost all actin was recovered in the cytosolic fraction. There was no statistically significant difference in Coro1 association to the membrane fraction in the absence (35.7 ± 9.6%) or presence (27.1 ± 11.7%) of LatB, indicating that the association of Coro1 to platelet membranes is independent of its association with actin. In these experiments, probing for the cytosolic marker in resting platelets spleen tyrosine kinase (Syk) and the membrane marker CD36 confirmed that each fractionation was free from cross-contamination.

We next characterized the association of Coro1 to the actin cytoskeleton. Resting human platelets were lysed in the presence of Triton X-100 and separated into soluble (containing G-actin) and insoluble (containing F-actin) fractions by centrifugation at low and high speeds followed by immunoblot analysis of the fractions (Figure 1C)[21]. Under these conditions, actin is distributed as approximately 60% soluble and 40% insoluble. At low speed, almost 60% of Coro1 was present in the Triton X-100 insoluble pellet, which contains large crosslinked actin filaments. Treatment with LatB, which efficiently depolymerized actin filaments, solubilized most of the Coro1, indicating that Coro1 in the LS pellet is predominantly associated with F-actin.

At high speed nearly all the Coro1 was recovered in the Triton X-100 insoluble pellet, which contains short actin filaments, even upon treatment with LatB, indicating that the association of Coro1 to the HS pellet is independent of an association with short actin filaments. We investigated the behavior of profilin and cofilin, two proteins involved in actin filament turnover. Profilin was recovered in the supernatants at both LS and HS, consistent with its role as monomeric actin-binding protein. Cofilin, which interacts with F-actin in addition to G-actin, was observed in HS and LS pellets and was removed from the LS pellet upon actin depolymerization, but not completely from the HS pellet. Coro1, as well as part of cofilin, may be associated with membranes or membrane proteins independently of actin, as suggested by the presence of a fraction of the membrane protein and lipid rafts component CD36 [22]. Syk was used as Triton X-100 soluble fraction marker to confirm that isolation of cytoskeleton fraction was clean.

To verify whether mouse Coro1 behaves similarly to human Coro1, we carried out subcellular and Triton-X100 fractionations as above and observed a similar distribution in membrane and cytosol fractions as well as in Triton-X100 supernatant and pellet fractions of mouse platelets (Supplemental Figure 3A, B).

### Localization of Coronins in Platelets

**Figure 2.**
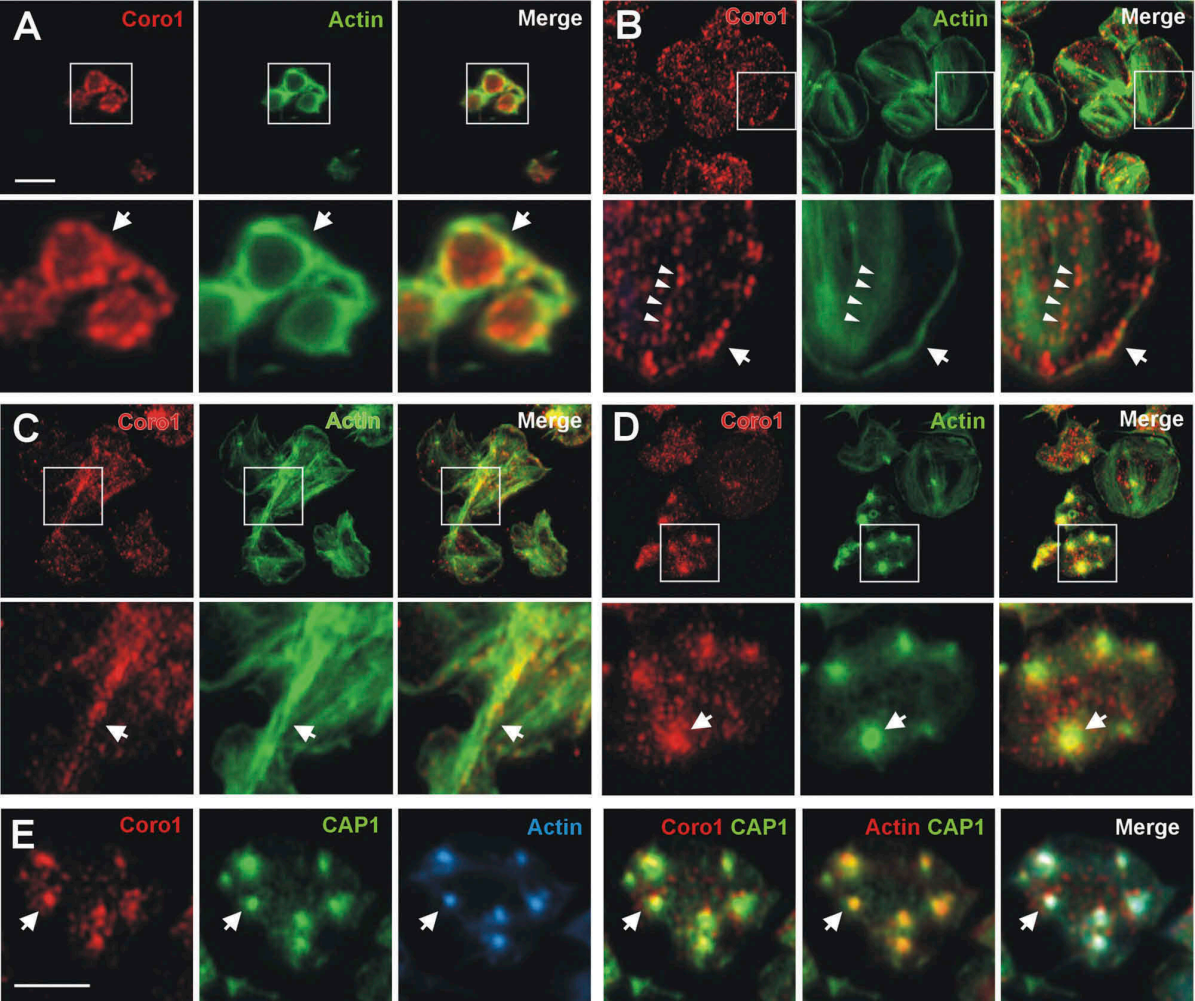
Subcellular localization of Coro1. Human platelets were fixed in suspension with paraformaldehyde and spun on poly-L-lysine coated coverslips (A) or were allowed to spread on 100 µg/ml fibrinogen (B, D) or collagen (C) coated coverslips and fixed with paraformaldehyde. For A, B, C and D cells were immunostained with an anti-Coro1 antibody followed by an Alexa568-coupled secondary antibody (red) and counterstained with FITC-phalloidin for filamentous actin (green). For E platelets were treated with 100 nM PGI2 at 37°C 5 min prior to fixation in order to increase the proportion of cells displaying actin nodules. Platelets were then immunostained with anti-Coro1 and anti-CAP1 antibodies followed by Alexa568 and Alexa488-coupled secondary antibodies, respectively (red and green), and counterstained with Alexa680-phalloidin for filamentous actin (blue). Actin color has been changed to red in the double staining panel with CAP1 for better visualization. Images were acquired with a fluorescence microscope equipped with a structured illumination attachment and deconvolved. Magnified regions are indicated with a square. Arrows point at regions of interest: cell cortex (A, B), actin filaments (C), actin nodules (D, E). Arrowheads in B point at Coro1 along stress fibers. Scale bars 5 µm. The scale bar on A applies to B, C, and D.

We used immunostaining and fluorescence microscopy to study the distribution of Coro1, 2 and 3 in human and mouse platelets. In resting platelets in suspension, which are predominantly discoid, the distribution of Coro1 is punctate, very often accumulating at the cell cortex, where it colocalizes with F-actin (Figure 2A). In platelets spread on fibrinogen Coro1 displays a predominantly punctate diffuse distribution, with frequent instances of discontinuous accumulation in cortical regions, where the protein co-localizes with F-actin (Figure 2B, arrows). Under these conditions, Coro1 does not display a striking pattern of association to stress fibers, although in some cases puncta appear to align with stress fibers (Figure 2B, arrowheads). Platelets spread on collagen fibers extend more broadly and present more prominent stress fibers. Under these conditions we often see Coro1 accumulating along with thick actin cables in a discontinuous manner (Figure 2C). Coro1 conspicuously accumulates at actin nodules (Figure 2D), where it colocalizes with CAP1, which we have described previously as a component of actin nodules (4). Both Coro1 and CAP1 display a broad pattern of accumulation compared to the sharper pattern of F-actin (Figure 2E).

**Figure 3.**
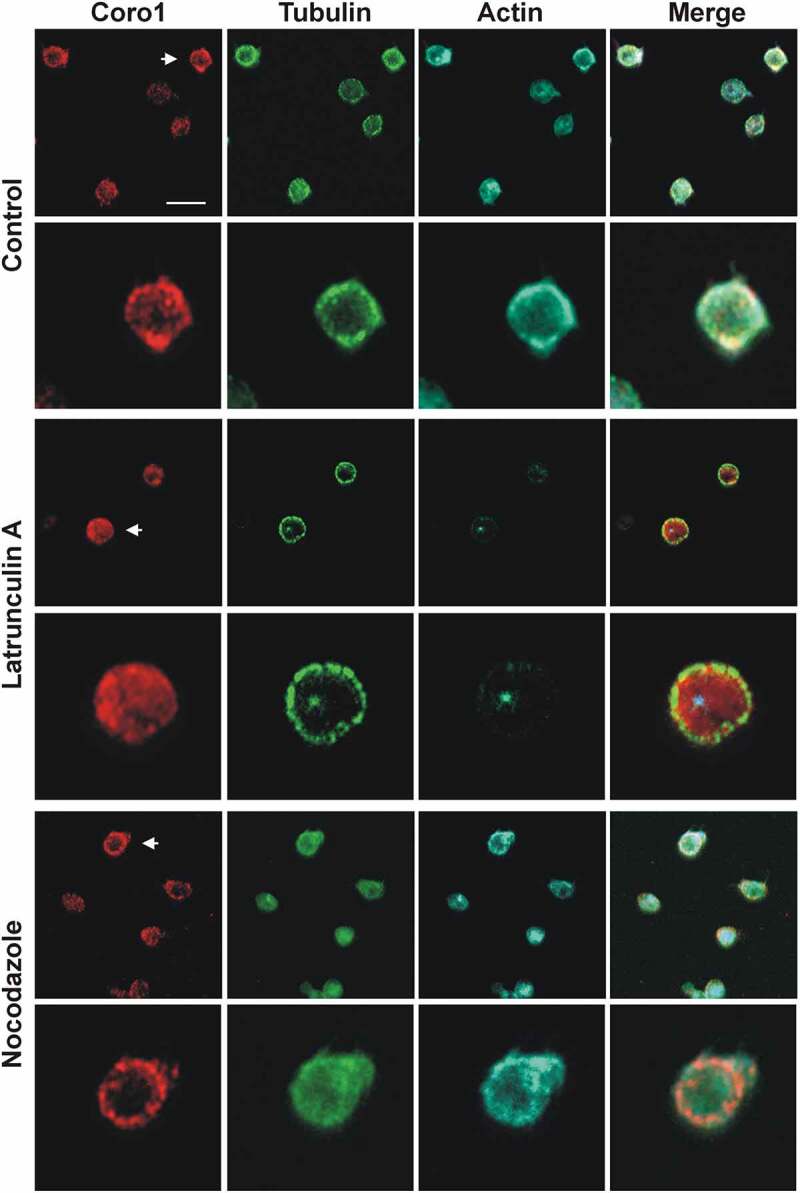
Subcellular localization of Coro1 upon disruption of the actin and tubulin cytoskeletons. Human platelets were incubated with 3 µM LatB or 10 µM nocodazole for 30 min, fixed in suspension with paraformaldehyde and spun on poly-L-lysine coated coverslips. Cells were immunostained with anti-Coro1 and anti-tubulin antibodies followed by Alexa568 and Alexa488-coupled secondary antibodies, respectively (red and green), and counterstained with Alexa680-phalloidin for filamentous actin (blue). Images were acquired with a fluorescence microscope equipped with a structured illumination attachment and deconvolved. Arrows indicate the magnified cell shown in the second row of each treatment. Scale bar 5 µm.

Coronins have been proposed as organizers of the actin, microtubule and intermediate filament systems [23]. We investigated the effect of disrupting the actin or microtubule cytoskeletons on Coro1 localization in platelets in suspension (Figure 3). LatB treatment resulted in almost complete disappearance of filamentous actin and loss of Coro1 cortical accumulation while the microtubule ring was intact. By contrast, treatment with the microtubule depolymerizing drug nocodazole resulted in the dispersal of the microtubule ring but intact cortical accumulation of Coro1 and actin. This indicates that Coro1 accumulation at the platelet cortex is primarily dependent on F-actin.

**Figure 4.**
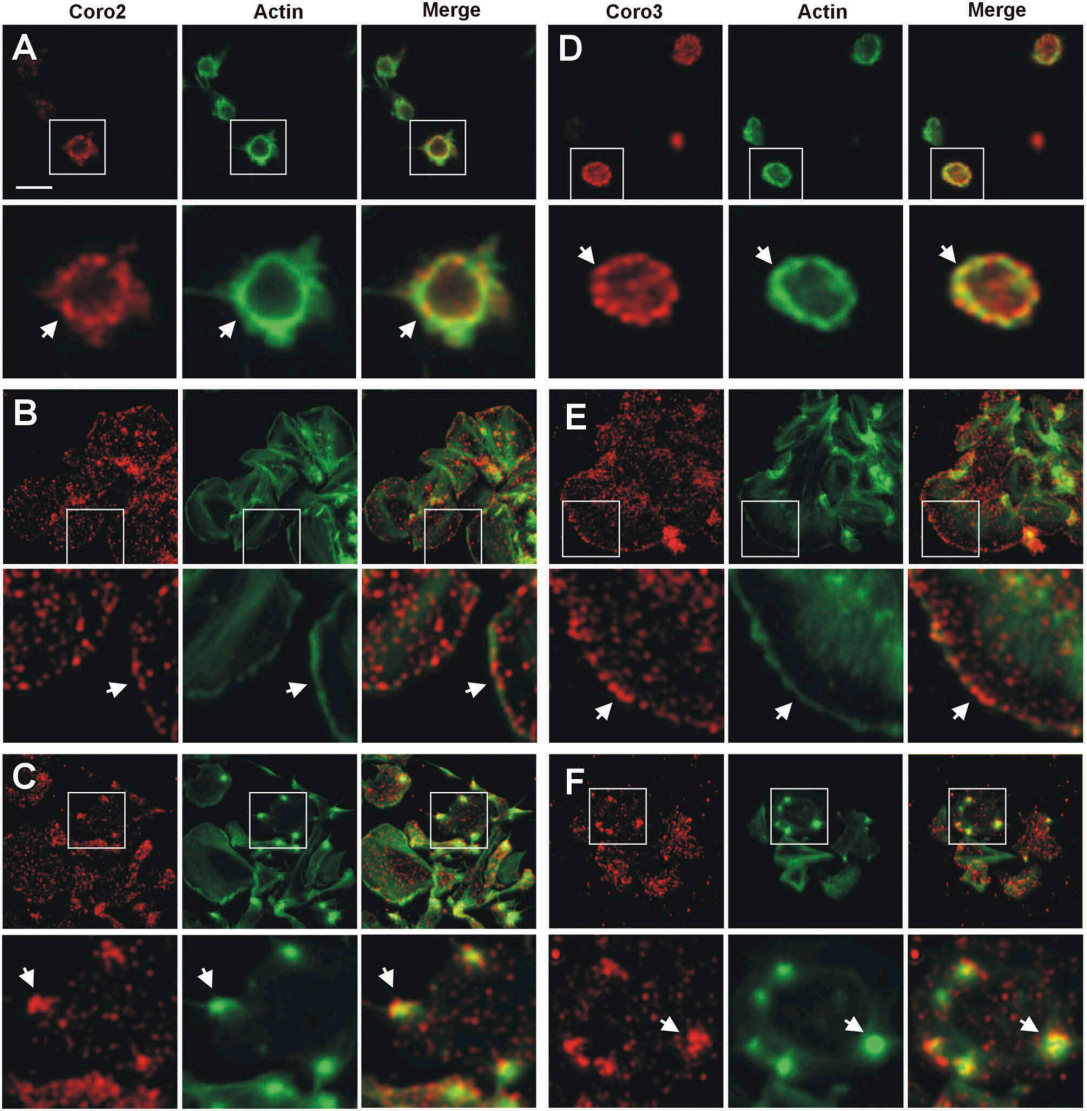
Subcellular localization of Coro2 and 3. Human platelets were fixed in suspension with paraformaldehyde and spun on poly-L-lysine coated coverslips (A, D) or were allowed to spread on 100 µg/ml fibrinogen coated coverslips (B, E, C, F) and fixed with paraformaldehyde. Cells were immunostained with an anti-Coro2 or Coro3 antibody followed by an Alexa568-coupled secondary antibody (red) and counterstained with FITC-phalloidin for filamentous actin (green). Images were acquired with a fluorescence microscope equipped with a structured illumination attachment and deconvolved. Magnified regions are indicated with a square. Arrows point at regions of interest: cell cortex (A, B, D, E), actin nodules (C, F). Scale bar 5 µm.

Coro2 and Coro3 display patterns of localization very similar to Coro1. In platelets, in suspension, both proteins show a punctate pattern, often enriched at the cell cortex and colocalizing with F-actin (Figure 4A, D). When spread on fibrinogen the pattern of localization of Coro2 and Coro3 was relatively uniformly dotty, with frequent discontinuous accumulation at the cell cortex (Figure 4B, E). This pattern of cortical enrichment was less apparent with Coro2, which in general gave an overall weaker staining than Coro3. Both coronins also accumulated and colocalized with F-actin at actin nodules (Figure 4C, F). Taking into account the weaker staining of Coro2, its accumulation at actin nodules appeared relatively more intense than that of Coro3.

In mouse platelets spread on fibrinogen the localization of Coro1, 2 and 3 resembled that of the respective human counterpart, with a predominantly punctate pattern and frequent discontinuous accumulation and colocalization with F-actin at the cortex (Supplemental Figure 3C).

### Class I Coronins Co-immunoprecipitate and Colocalize in Platelets

**Figure 5.**
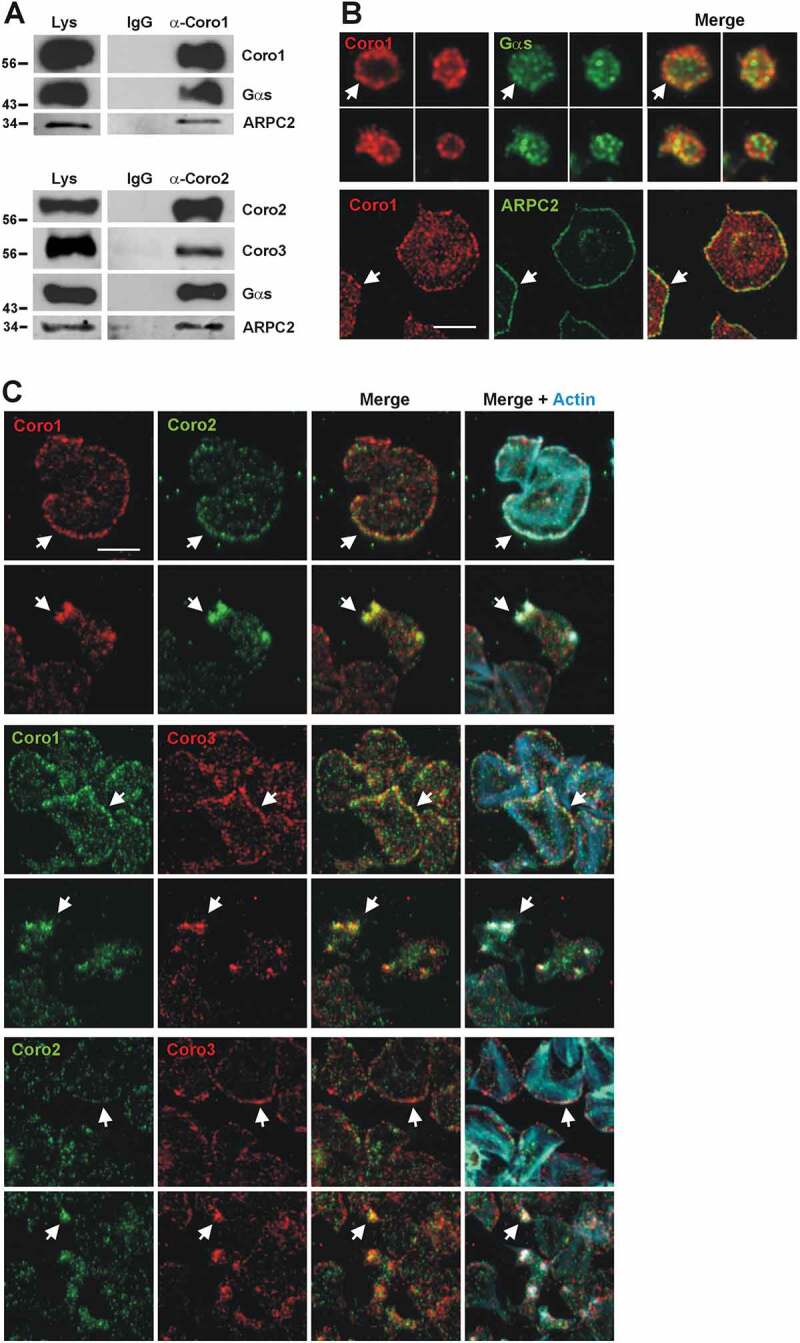
Coronins exist in complexes with each other and with Gαs and the Arp2/3 complex. (A) Human platelet lysates were subject to immunoprecipitation with Coro1 or Coro2-specific antibodies. The same species of the total immunoglobulin G (IgG) was used as a control. Protein complexes were examined by Western blot for the presence of the indicated proteins. (B) Colocalization of Coro1 with Gαs and ARPC2. For Gαs platelets were fixed in suspension with paraformaldehyde and spun on poly-L-lysine coated coverslips. For ARPC2 platelets were allowed to spread on 100 µg/ml fibrinogen coated coverslips and fixed with paraformaldehyde. Cells were immunostained with anti-Coro1 and anti-ARPC2 or anti-Gαs antibody followed by Alexa568 or Alexa488-coupled secondary antibodies, respectively (red and green). Images were acquired with a fluorescence microscope equipped with a structured illumination attachment and deconvolved. Arrows point at regions of apparent colocalization. Scale bar 5 µm. (C) Coronins colocalize with each other. Platelets were allowed to spread on 100 µg/ml fibrinogen coated coverslips, fixed with paraformaldehyde, immunostained with the indicated coronin antibodies followed by Alexa568 or Alexa488-coupled secondary antibodies, respectively (red and green), and counterstained with Alexa680-phalloidin for filamentous actin (blue). Images were acquired as in (B). Arrows point at regions of interest: cell cortex (upper panels), actin nodules (lower panels). Scale bar 5 µm.

Coro1 has been reported to interact with the Gαs subunit of heterotrimeric G proteins and stimulate the cAMP/PKA pathway in murine excitatory neurons and loss of Coro1 results in neurobehavioral defects [24]. To explore whether this interaction reproduces in platelets we performed immunoprecipitation experiments in human platelet lysates and found that Coro1 is able to co-immunoprecipitate Gαs (Figure 5A, upper panel). Upon co-immunoprecipitation of Coro2, Gαs was also retrieved in the immunocomplexes along with Coro3 (Figure 5A, lower panel). In support of Gαs forming complexes with coronins, we immunostained platelets in suspension for Coro1 and Gαs. Coro1 showed the characteristic discontinuous cortical accumulation, whereas the distribution of Gαs was punctate and uniform. We observed, however, instances of co-localization of both proteins at the cell cortex (Figure 5B). In these experiments, ARPC2, a subunit of the Arp2/3 complex, was found in the immunocomplexes and partially colocalizing with Coro1 at the cell cortex of spread platelets, compatible with the already reported interaction of the Arp2/3 complex with class I coronins (Figure 5B) [25–27]. To further investigate this colocalization we studied the effect of inhibiting the Arp2/3 complex on Coro1 localization (Supplemental Figure 4). We treated platelets with the Arp2/3 complex inhibitor CK-666 at a range of concentrations spanning three orders of magnitude (0.5, 5 and 50 µM), stimulated them with thrombin and allowed them to spread on fibrinogen for 45 min. Without thrombin stimulation, most untreated platelets adopted a spiky morphology with numerous actin nodules but responded to thrombin with an extended round morphology and clear enrichment of ARPC2 at the cortex, with Coro1 often accumulating at the cortex too. The lower concentration of CK-666 had little effect on ARPC2 and Coro1 localization. Increasing concentrations of the inhibitor resulted in a high proportion of non-spread round platelets in the absence of thrombin stimulation, however the cells responded to thrombin. While 5 µM CK-666 still resulted in round well-spread platelets, with 50 µM CK-666 most platelets adopted an irregular shape with a few filopods, consistent with the inhibited formation of lamellipodia [28]. The cortical accumulation of ARPC2 persisted upon treatment with CK-666, consistent with the fact that CK-666 stabilizes the inactive state of the Arp2/3 complex, but does not prevent its binding to actin filaments [29], however Coro1 cortical accumulation was largely lost.

The similar patterns of subcellular localization of all three class I coronins suggest that they might be performing similar functions and may co-localize and participate in complexes with each other. In fact, as indicated above, Coro2 and Coro3 co-immunoprecipitate. To investigate the extent of co-localization of the three-class I coronins in platelets we performed a combination of double stainings (Figure 5C). These studies revealed that while all three coronins accumulate at the cell cortex, some extent of co-localization was apparent in all combinations, although in the case of Coro1 and Coro3 colocalization seemed clearer. A clear pattern of colocalization was observed in actin nodules in every combination of coronin immunostainings.

### Translocation of Coronins upon Platelet Stimulation

**Figure 6.**
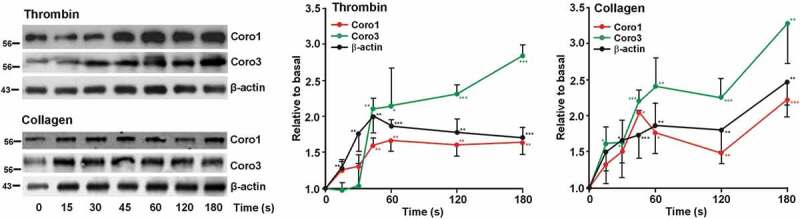
Dynamics of Coro1 along with F-actin in the Triton X-100 insoluble pellet of platelets stimulated with thrombin or collagen. Washed human platelets were stimulated with thrombin (0.1 U/ml) or collagen (50 µg/ml) at the indicated time points and lysed immediately in 1% Triton X-100 lysis buffer. Triton insoluble pellets were prepared by low-speed centrifugation (15,600 × g for 15 at 4°C), run on SDS-PAGE and subjected to Western blot analysis with anti-Coro1, Coro3 and β-actin antibodies. Densitometry values are expressed as means ± SEM of 3–7 experiments *P < .05, **P< .01, ***P < .001 relative to basal, student’s t-test.

Stimulation with strong agonists typically provokes a rapid increase in actin polymerization that can be monitored on time by analyzing the amount of actin in the LS detergent-insoluble pellet. Both upon thrombin and collagen stimulation the proportion of actin in the LS pellet rapidly increased to a twofold peak at 60 s and remained elevated afterward (Figure 6). We explored the effect of those agonists in the association of Coro1 and 3 to the LS pellet. Platelets were stimulated with 0.1 U/ml thrombin or 50 μg/ml collagen and the reaction was stopped with lysis buffer at various time points up to 3 min. We observed a statistically significant time-dependent increase in the proportion of both Coro1 and 3 in the LS pellet that roughly paralleled that of actin (Figure 6). In mouse platelets both actin and Coro1 behaved similarly to their human counterparts in response to thrombin and collagen, although Coro1 appeared to peak earlier (at 15 s) (Supplemental Figure 3D).

**Figure 7.**
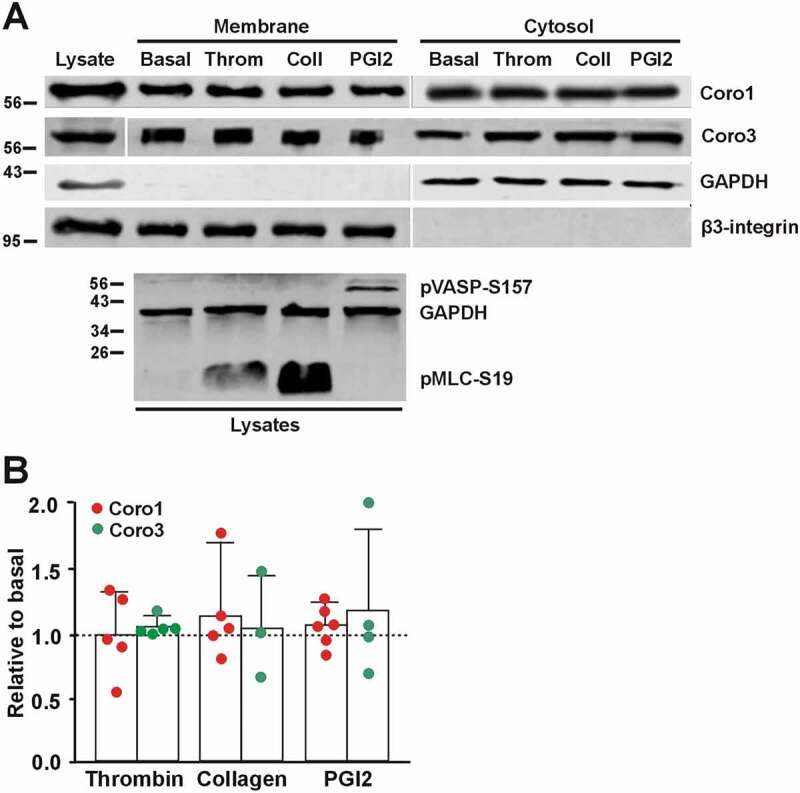
Coronins do not translocate upon platelet stimulation. (A) Washed human platelets (8 × 10^8^/ml) were treated with 1 mM EGTA, 10 nM indomethacin and 2 U/ml apyrase for 20 min at 37°C to prevent aggregation. They were then treated with 0.1 U/ml thrombin, 50 µg/ml collagen or 100 nM PGI2 for 1 min at 37°C prior to lysis and subcellular fractionation. Fractions were normalized by volume and resolved on 12% SDS-PAGE, blotted onto PVDF membrane and probed with antibodies for Coro1 and Coro3. Integrin β3 was used as a membrane marker and GAPDH as a cytosolic marker. The phosphoproteins pMLC-S19 and pVASP-S157 were used as markers of the effects of thrombin/collagen and PGI2, respectively, and GAPDH as a loading control. (B) Membrane-associated Coro1 and Coro3 upon stimulation were quantified by densitometry, normalized to integrin β3 and expressed relative to the respective coronin in the basal membrane fraction. Data represent the mean ± SEM of 3–6 independent experiments. No statistically significant differences were found relative to basal for any coronin using Mann-Whitney U and Kruskal-Wallis tests.

As shown above, one-third of Coro1 is membrane associated. To investigate whether exposure to various stimuli would affect this pattern of distribution we treated platelets in suspension with 0.1 U/ml thrombin, 50 μg/ml collagen or 100 nM PGI2 and subjected them to subcellular fractionation followed by Western blot analysis. We did not observe any significant change in the proportion of Coro1 or Coro3 upon any of the treatments (Figure 7).

## Discussion

In this study, we present immunological evidence of the presence of members of the coronin family in human and mouse platelets. We show that class I coronins Coro1, 2 and 3 are abundant in platelets from both species, whereas expression of Coro7 is comparatively very low. Our results are in very broad agreement with data from proteomics and transcriptomic studies (Supplemental Table 1 and 2), with the caveat that western immunoblot results are not suitable for quantitative comparisons due to the fact that all the antibodies we have used were raised against fragments of human coronins; therefore, we expect them to have different affinities for the corresponding mouse protein. In addition, the affinities of the antibodies are also expected to vary among the different coronins of the same species.

At 53,600 copies per platelet, class I coronins taken together are among the most abundant proteins in human platelets [30], and appear to be approximately 2.5 times more abundant in mouse platelets [31]. Coro2 exists in less copies per platelet than Coro1 and 3 in both human and mouse, which might explain the weaker signal we usually observe in immunostainings for this coronin. While Coro1 and 3 are present in similar copy numbers in human platelets, Coro1 is considerably more abundant than Coro3 in mouse platelets [30,31]. Interestingly, proteomics and transcriptomics studies reveal a lack of correlation of protein and transcript levels: Coro3 mRNAs are present at considerably high levels in human platelets, whereas Coro1 mRNAs are present at very low levels in mouse platelets [32].

Coro7 is the least abundant coronin in platelets, with an estimated 760 and 3571 copies in human and mouse platelets, respectively [30]. Our western blot data clearly reflect this difference in abundance between species. Some proteomics and transcriptomics studies fail to identify Coro7, probably due to technical limitations [33,34], or to expression lying below the cutoff set for a gene to be included in the list of expressed genes [35,36]. A significant proportion of Coro7 associates with Golgi membranes [19,20]. Usually, very little Golgi is present in mature platelets, explaining the relatively very low abundance of this isoform, which we speculate might have a role in platelet maturation by regulating Golgi-related processes. Based on transcriptomics studies, Coro4, 5 and 6 do not appear to be expressed to significant levels in human and mouse platelets and consequently remain undetectable in proteomics studies (Supplemental Table 1 and 2).

In platelets, approximately 36% of Coro1 is recovered in the membrane fraction and this association is independent of the actin cytoskeleton, as indicated by its persistence after LatB treatment. This is in broad agreement with a study in J774 macrophages, where Gatfield et al. found 20% of Coro1 associated with membranes [8]. Also, 40% of Coro3 had previously been found associated with membranes [7]. We are not aware of any study that formally addresses the subcellular distribution of Coro2, but this coronin is required for endosome fission, therefore some extent of membrane association is expected [37]. Membrane association has been reported for the *Plasmodium falciparum* homolog too [38,39], and therefore seems to be a common feature of coronins. Several mechanisms might account for the membrane association of coronins, most notably their ability to directly bind PI(4,5)P2 [39,40], but interactions with other membrane-associated proteins are also likely to contribute, for example, Rac1 and Gαs with Coro1 and Rab27a with Coro3 [24,41,42]. None of the stimuli we have tested in platelets produces a noticeable translocation of Coro1 or 3 between the membrane fraction and the cytosol, suggesting that either translocation is not required for coronin function or the amount that translocates is below the levels detectable with our techniques.

Sixty percent of the Coro1 fractionates in the LS detergent-insoluble pellet of platelets, predominantly associated with F-actin. A similar behavior has been described for this coronin in J774 macrophages [8]. The proportion of Coro1 in the LS pellet increases rapidly upon stimulation with strong agonists and Coro3 shows a comparable behavior, consistent with the role of class I coronins in actin filament remodeling in platelets. By contrast, virtually all the Coro1 was recovered in the HS pellet of platelets and this association was not disrupted by LatB, indicating that it is independent of actin. This may represent Coro1 associated with membrane-containing structures like lipid rafts, along with a fraction of cofilin. Coro3 too has been reported as abundant in the HS pellet of HaCat cells, from where it is partially extracted in the presence of LatB [26].

Coro1, 2 and 3 display a similar localization in human platelets, with a diffuse punctate cytoplasmic localization and a discontinuous enrichment at the cortex of both suspended and spread platelets, where it co-localizes with F-actin and, in the latter, the Arp2/3 complex. This pattern is similar to the reported localization of Coro1 in macrophages and lymphocytes, [8,13,43,44] as well as in unicellular organisms like *Dictyostelium discoideum, Trichomonas vaginalis* and *Plasmodium falciparum* [39,45,46], but Coro1 is also recruited at phagosomes in macrophages and neutrophils [27,43]. Coro2 and 3 have been shown to display a diffuse cytosolic localization with enrichment at peripheral protrusions in a variety of cells, like DRG neurons, lung endothelial cells, fibroblasts, HEK cells, oligodendrocytes, HaCat cells, and Pop10 hepatocarcinoma cells [7,25,26,47–49]. The accumulation of Coro1 at the cell cortex seems to be dependent on the activity of the Arp2/3 complex and is evident only in spread platelets morphologically compatible with the presence of lamellipods. We observed Coro1 localizing in a discontinuous pattern at stress fibers, more clearly in platelets spread on collagen, a matrix protein that usually leads to the formation of more robust actin cables compared to fibrinogen. Localization of class I coronins at stress fibers has been very seldom reported and might indicate sites of active remodeling under specific circumstances [23]. Mouse class I coronins showed a pattern of predominantly diffused cytoplasmic distribution with some cortical accumulation, similar to their human counterparts and in agreement with a recent report that addressed the immunolocalization of Coro1 in mouse platelets [5].

A localization of class I coronins in actin nodules has not been reported before. These podosome-related structures consist of a core rich in actin and Arp2/3 complex core surrounded by a ring rich in focal adhesion molecules like talin and vinculin [50]. They are usually visible during early adhesion and spreading [51]. We have recently shown that CAP1, a protein involved in recycling of actin monomers, is also a component of the actin nodule ring [4], therefore we speculate that coronins too might contribute to the actin filament turnover of these highly dynamic structures.

Class I coronins are reported to exist as homo-oligomers and there is no evidence for the formation of hetero-oligomers [7,8,25]. The colocalization of class I coronins to the same structures in platelets suggests that they might be part of large complexes containing more than one isoform, as demonstrated by the ability of Coro2 to co-immunoprecipitate Coro3. This is in contrast to the report of Cai et al. (2005) that failed to observe a co-immunoprecipitation of Coro2 with any other coronin in a fibroblast cell line [25], however large-scale interactome studies have identified class I coronins as part of the same complexes [52,53].

In summary, we provide evidence that class I coronins are abundant cytoskeleton regulators in platelets, where they may play roles in organizing the cortical cytoskeleton upon adhesion and spreading, consistent with the emerging role of coronins as integrators of extracellular signals with actin remodeling. The fact that class I coronins co-localize and different isoforms might participate in the same complex strongly suggests a high extent of functional overlap and would explain the mild phenotype of platelets lacking Coro1 [5]. Functional overlap is expected to occur in most white blood cell types, where all three class I coronins appear to be expressed simultaneously (Supplemental Figure 5). Further studies on animal models lacking one or more class I coronins will be required to elucidate the unique and shared roles of these proteins in platelet function.
